# Use of the amber teething necklace by the child population: risks *versus* benefits

**DOI:** 10.1590/1984-0462/2022/40/2020412IN

**Published:** 2022-05-27

**Authors:** Ana Lídia Soares Cota, Emilly Alves da Silva, Nicole Beatriz Barros de Sá Freitas, José Sarmento Lins Irmão Bisneto, Gabriella Marinho Buriti, Júlia Quintella Lessa Maia Valente, Mariana Alencar Nemezio

**Affiliations:** a Centro Universitário Tiradentes, Campus Maria Amélia Uchôa, Maceió, AL, Brazil.

**Keywords:** Amber, Deciduous teeth, Strangulation, Local symptoms, Âmbar, Dente decíduo, Estrangulamento, Sintomas locais

## Abstract

**Objective:**

Based on scientific evidence, the objective of the present study is to report the possible risks and benefits of the amber teething necklace for children who use it.

**Data source:**

This is an integrative literature review, carried out based on the following guiding question: “Does the amber teething necklace have therapeutic properties that justify its usage during tooth eruption?”. The consulted databases were LILACS (Latin American and Caribbean Health Sciences Literature) and PubMed (National Center for Biotechnology Information), with the following descriptors: “Amber,” “Deciduous teeth,” “Strangulation,” and “Local symptoms.”

**Data synthesis:**

A total of five scientific articles were selected, which indicates an insufficient basis regarding the benefits associated with the use of the amber teething necklace. Conversely, there is a convergence regarding the possibility of health risks such as strangulation, asphyxiation, and swallowing of beads.

**Conclusions:**

Health professionals should discourage the use of the amber teething necklace by children insofar more studies on the topic are carried out.

## Resumo

**Objetivo:**

Com base em evidências científicas, a proposta do presente trabalho é relatar os prováveis riscos e benefícios do uso do colar de âmbar pela população infantil.

**Fontes de dados:**

Trata-se de uma revisão integrativa da literatura, realizada a partir da elaboração da questão norteadora: “O colar de âmbar apresenta propriedades que justifiquem seu uso durante a erupção dentária?’’. As bases de dados utilizadas foram LILACS (Literatura Latino-americana e do Caribe em Ciências da Saúde), PubMed (National Center for Biotechnology Information), por meio dos descritores: Âmbar; Dentes decíduos; Estrangulamento; Sintomas locais.

**Síntese dos dados:**

Após a busca dos dados, foram selecionados cinco artigos, os quais apontam insuficiente embasamento científico que comprove os benefícios relacionados ao uso do colar de âmbar. Em contrapartida, existe uma convergência quanto à possibilidade de riscos à saúde, como estrangulamento, asfixia e deglutição das pedras.

**Conclusões:**

Conclui-se que os profissionais da saúde devem desencorajar o seu uso por crianças até que pesquisas clínicas bem delineadas sejam disponibilizadas.

## INTRODUCTION

During the eruption of primary teeth, around six months of life, local manifestations in the tissues surrounding the teeth and/or systemic manifestations are commonly reported by family members. Among these, the presence of gingival itching and inflammation, loss of appetite, increased thumb sucking or putting objects in the mouth, hypersalivation, low-grade fever, irritability, diarrhea, and insomnia stand out. However, it is worth emphasizing that the occurrence of systemic biological manifestations associated with the tooth eruption process is a debate that has not yet been elucidated by the scientific community. Thus, these manifestations are still considered as social beliefs, as symptoms, such as fever and diarrhea, may be related to the simple fact that children put their dirty hands into their mouths to relieve gingival itching.^1–3^

Although tooth eruption is defined as a physiological and benign process, many parents and guardians of children tend to consult pediatricians and pediatric dentists seeking to access any treatment for minimizing or treating the symptoms. Currently, professionals have two types of therapeutic management. The non-pharmacological management should be the approach of choice and consists in pressuring the painful area of the mucous membrane with objects such as teethers made of solid silicone or frozen fruits and vegetables. Conversely, the pharmacological management is restricted to the systemic use of analgesics and/or topical agents such as local anesthetics with lidocaine-based medications.^4^

In mid-2011, the amber necklace emerged in the world market as a “natural” therapeutic alternative for the relief of local discomforts. Deemed as a bio-transmitter, amber stone spheres, which are used as a necklace or even bracelets and anklets, would release succinic acid through contact and heat. When absorbed by the skin, the acid would produce local analgesic and anti-inflammatory effects. Manufacturers recommend the continuous and prolonged use of the product to obtain the desired therapeutic effect.^5^

With the advent of social media and networks, numerous pieces of information have been published on virtual platforms without proper data legitimation. As a consequence, an excess of (mis)information is quickly and damagingly spread, thus instigating researchers and health authorities. Therefore, the objective of the present study is to evaluate scientific evidence about the use of the amber teething necklace and its risks and benefits for the child population, emphasizing its therapeutic function considering the clinical manifestations associated with tooth eruption.

## METHOD

This is an integrative literature review, a methodology that allows a careful analysis of previously published studies as a reference for conducting new research. By evaluating, interpreting, and synthesizing the results, it is possible to develop an explanation about a previously addressed topic.^6^ In the present study, the research theme was formulated based on the following guiding question: “Does the amber teething necklace have therapeutic properties that justify its usage during tooth eruption?”.

The databases consulted for data searching were LILACS (Latin American and Caribbean Health Sciences Literature), with descriptors extracted from the DeCS (Health Sciences Descriptors) platform, both in Portuguese and English languages: *Âmbar*; *Dentes Decíduos*; *Estrangulamento*; and *Sintomas Locais*, in Portuguese; and “Amber”; “Deciduous Teeth”; “Strangulation”; and “Local Symptoms”, in English. PubMed (National Center for Biotechnology Information) was also consulted using the same descriptors. Initially, a search of articles was conducted through the descriptors individually; then, they were crossed in pairs and trios using the Boolean operator “AND.”

**Figure 1.**
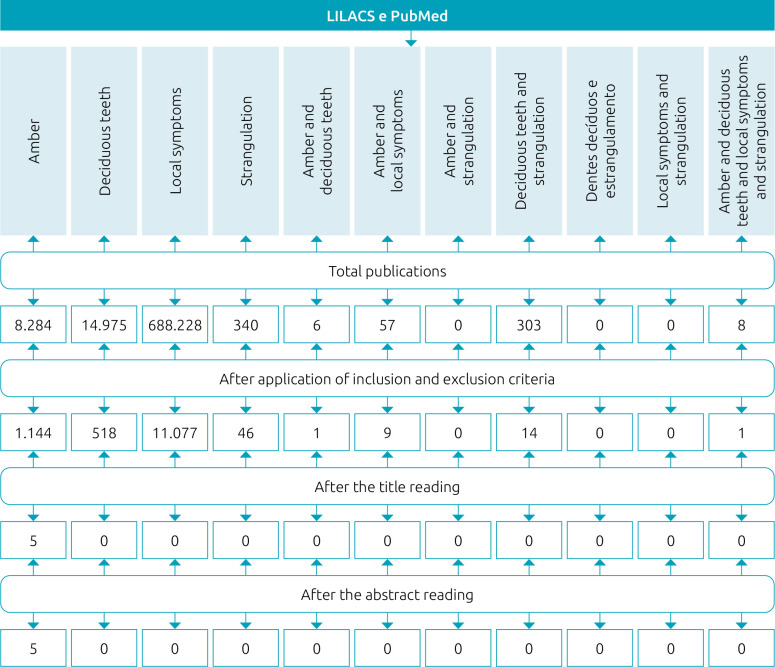
Flowchart illustrating the trajectory and search strategies of the study.

The inclusion criteria for sample selection consisted in articles published in Portuguese and/or English languages between 2015 and 2020, freely available in full, and which addressed the study theme after reading the titles and abstracts. Books, chapters of books, undergraduate thesis, and dissertations were excluded from the study. The trajectory and searching strategies used in the present study are illustrated in Figure 1.

## RESULTS

After reading the abstracts of the studies, five articles were selected for this research, and all of them were linked to the LILACS database. All articles were published in English and are characterized according to their type of study such as observational study, case report, and retrospective longitudinal study. The included articles derived from the descriptor “Amber” and originate from countries in the Americas such as Brazil, the United States of America, and Canada. The authors’ fields of knowledge were dentistry and medicine (Table 1).

**Table 1.** Description, in chronological order of publication, of the articles selected for the integrative review.

Authors (year)^reference^	Objective	Main results	Conclusion
Machet et al. (2016)^10^	To analyze the bacterial colonization on amber necklaces used by children during hospital appointments.	All necklaces analyzed had bacterial colonization. 32 different species were found, the most frequent being coagulase-negative staphylococci. In addition, 70.4% of parents consider risky using this necklace on their children.	Amber necklaces can be highly colonized, being contraindicated, as they are susceptible to bacterial infections.
Cox et al. (2017)^1^	To report an accidental infant strangulation case caused by the use of amber teething necklace.	A 4-month-old patient used an amber teething necklace while sleeping to decrease the discomfort caused by tooth eruption and woke up with petechiae on the face and neck, compatible signs with the diagnosis of accidental strangulation.	It is extremely important that health professionals who work with children make safe recommendations for those responsible, considering that amber teething necklaces present strangulation risks.
Soudek and McLaughlin (2018)^11^	To analyze the strength required to break amber necklaces, in accordance with the Standard Specification of the American Society for Testing and Materials.	Of the 15 collars tested, seven did not break until the strength of 15 pounds, and eight out of ten did not break at a force of 1.6 pounds (standardized by the ASTM – American Society of Tests and Materials).	The amber teething necklaces present risk of strangulation in young children, as they cannot open so easily with the minimum strength applied.
Nissen et al. (2019)^5^	To evaluate the efficiency of the amber teething necklace in treating symptoms of tooth eruption.	All amber stones submerged in saline buffered with pH 5.5 octanol phosphate did not release measurable succinic acid, except for light-colored spheres, which were divided into small fragments. In addition, treatment of macrophages with succinic acid did not reduce the release of any measured inflammatory cytokines and exhibited toxicity to cells in high concentrations.	There is no evidence that the active ingredient, succinic acid, contains anti-inflammatory properties and can be released from amber stones onto human skin.
Strieder et al. (2019)^8^	To determine Google users’ interest in information about the use of amber necklace in different countries over the years.	There has been an increase in interest from users, particularly since the year 2004, without the influence of monthly or quarterly seasonality. Most searches were about information to reduce discomfort, which demonstrates the perpetuation of beliefs associated with tooth eruption.	There is great interest of Google users regarding the use of the amber necklace as a solution for the relief of tooth eruption symptoms in several countries.

## DISCUSSION

Tooth eruption comprises the period from the initial formation of the tooth to its final position in the dental arch. On the days prior to the appearance of teeth in the oral cavity, characterized by the process of rupture of gingival tissues, some local signs and symptoms may occur.^3,7^ What is known to date is that regardless of the discomforts, they must not be disregarded.^2^ Based on this observation, treatment often requires the performance of a multidisciplinary health team. Thus, depending on children’s clinical condition, it is healthy and prudent on the part of pediatric dentists to refer them to the pediatrician to rule out any pathology and ensure an adequate therapeutic strategy.

Within this context, the study object of this research, the amber teething necklace, has gained prominence, particularly in social media, as a therapeutic alternative for relieving the symptoms associated with the eruptive process of teething.^8^ Machet et al. associate the growing interest in the product with the population’s search for naturopathic approaches in preference to conventional medicine. Overall, social media leads individuals to believe in news and (un)truthful pieces of information.^8–10^

In a research conducted by Strieder et al., the authors evidenced that parents constantly access the internet to find ways to minimize discomforts in the oral cavity of their children. However, this behavior often leads to erroneous beliefs and knowledge with no scientific evidence. The authors emphasize that, in Brazil, the marketing of the amber necklace has been boosted by an Instagram post made by the Brazilian model Gisele Bündchen, in which her daughter appeared using the accessory. Since then, the reach of digital media and its influence on boosting the sales of products were ratified, which are not always effective.^8^

According to results obtained by Nissen et al., there is no evidence of the release of intact succinic acid, a natural compound present in amber, to be absorbed by the human skin. Even if it was absorbed, it could not produce anti-inflammatory effects by inflammation mediators, considering the need for a much greater heat than that caused by the simple contact between the stone beads and the child’s body. This refutes the information presented by manufacturers of the product.^5,9,10^

In addition to the lack of evidence concerning the clinical effectiveness of the amber necklace, its use still implies imminent risks for the child’s health such as choking, strangulation, swallowing of the stone beads, and bacterial infections in the skin and/or in the oral cavity. In this sense, Machet et al.^9^ observed that these necklaces are associated with bacterial colonization on their surface, containing non-pathogenic bacterial species, in contact with healthy children; however, with the potential of causing infections in immunocompromised individuals.

The Standard Specification for Consumer Product Safety for Children’s Jewelry advocated by the American Society for Testing and Materials (ASTM),^11^ tested the amber necklace clasp and found that in 8 out of 10 necklaces, a mean force of 1.6 pounds was required to open the clasp, and in 8 out of 15, a force greater than 15 pounds was required to do so. Such force is sufficient to obstruct a small child’s airway when opening the clasp. The use of amber stone spheres in bracelets and anklets also presents risks, such as tourniquet, due to interruption of blood flow of the limbs in which they are worn.^8^ Supporters of its use claim that, even under considerable pressure, the necklace is more likely to break than to strangle any child. In Canada and in the United States of America, many stores sell the product based on this premise, but manufacturers are encouraged to test the product in advance. Nevertheless, in practice, this is not routinely carried out by the traders.^1^

The study of Nissen et al.^5^ demonstrated that only 8% of parents were aware of the risks related to the amber necklace usage. Notwithstanding, many of them remained convinced that “the benefits outweigh the risks.”^5^ Hence, they assumed that the possible and reported therapeutic effect could be subjective and stem from a placebo effect, which would soothe parents and guardians simply because it is an available option.^9^ The commercialization of goods through virtual and physical stores of children’s products has also exposed consumers to the purchase of necklaces made with synthetic stones, adding the risk of triggering allergic reactions.

Notorious medical associations, deemed as worldwide references in the fields of Pediatrics and Pediatric Dentistry, such as the Canadian Association of Emergency Physicians, the Brazilian Association of Pediatric Dentistry, the Brazilian Society of Pediatrics, and the American Academy of Pediatrics, advise parents not to use accessories containing amber stones on their children as well as no necklaces, strings, ribbons, or chains worn around the neck of children under three years of age.^1,11,12^ The Food and Drug Administration (FDA) strongly emphasizes this contraindication, considering reports of deaths from strangulation and choking from wearing the amber necklace.^1^ Finally, some authors point out that if parents and guardians still choose to use the amber necklace, its usage must be supervised and only during the day, removing it while the child is sleeping.^9–11^

The present review highlights the small number of scientific publications available on this topic, in addition to the predominance of descriptive studies, with a low level of evidence. Taking these limitations into consideration, the authors emphasize the need for conducting experimental clinical studies to reach a more elaborate debate on the topic.

Therefore, amber necklace has scientifically-proven more risks than evidence of its effectiveness in improving tooth eruption discomforts. The present results allow inferring that, up to date, health professionals should contraindicate its use in children, as there are no well-defined clinical research on the benefits of using this necklace, in addition to the doubtful association between systemic signs and symptoms and the tooth eruption process.
